# Real-Time Measurement of Cellobiose and Glucose Formation during Enzymatic Biomass Hydrolysis

**DOI:** 10.1021/acs.analchem.1c01182

**Published:** 2021-05-20

**Authors:** Hucheng Chang, Lena Wohlschlager, Florian Csarman, Adrian Ruff, Wolfgang Schuhmann, Stefan Scheiblbrandner, Roland Ludwig

**Affiliations:** † Biocatalysis and Biosensor Laboratory, Department of Food Science and Technology, BOKU—University of Natural Resources and Life Sciences, Muthgasse 18, 1190 Vienna, Austria; ‡ Analytical Chemistry—Center for Electrochemical Sciences (CES), Faculty of Chemistry and Biochemistry, Ruhr University Bochum, Universitätsstraße 150, 44780 Bochum, Germany

## Abstract

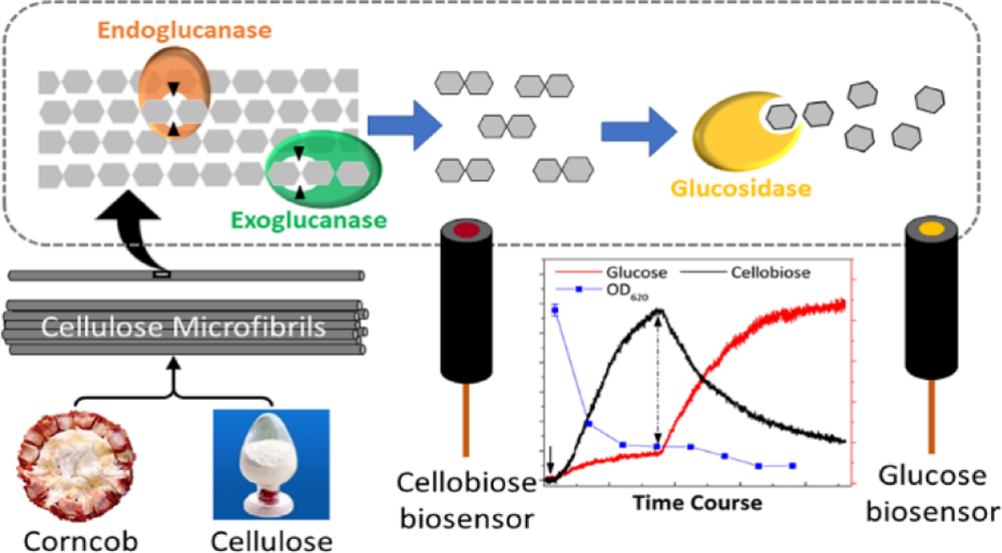

Enzymatic hydrolysis of lignocellulosic biomass for biofuel production relies on complex multi-enzyme ensembles. Continuous and accurate measurement of the released key products is crucial in optimizing the industrial degradation process and also investigating the activity and interaction between the involved enzymes and the insoluble substrate. Amperometric biosensors have been applied to perform continuous cellobiose measurements during the enzymatic hydrolysis of pure cellulose powders. The oxygen-sensitive mediators used in these biosensors restricted their function under physiological or industrial conditions. Also, the combined measurements of the hydrolysis products cellobiose and glucose require a high selectivity of the biorecognition elements. We employed an [Os(2,2′-bipyridine)_2_Cl]Cl-modified polymer and cellobiose dehydrogenase to fabricate a cellobiose biosensor, which can accurately and specifically detect cellobiose even in the presence of oxygen and the other main product glucose. Additionally, a glucose biosensor was fabricated to simultaneously measure glucose produced from cellobiose by β-glucosidases. The cellobiose and glucose biosensors work at applied potentials of +0.25 and +0.45 V versus Ag|AgCl (3 M KCl), respectively, and can selectively detect their substrate. Both biosensors were used in combination to monitor the hydrolysis of pure cellulose of low crystallinity or industrial corncob samples. The obtained results correlate with the high-performance liquid chromatography pulsed amperometric detection analysis and demonstrate that neither oxygen nor the presence of redox-active compounds from the lignin fraction of the corncob interferes with the measurements.

The enzymatic conversion of abundant, non-food lignocellulosic biomass into fermentable sugars is of great interest as a carbon-neutral, renewable feedstock base.^1,2^ One main challenge in the current biofuel industry is to overcome the reduction of the hydrolysis rate during the continuous reaction process.^3−5^ The origin of this slowdown is unclear, and both product inhibition or a clash of cascading enzyme reactions have been proposed.^6,7^ The fundamental understanding of biomass hydrolysis relies on comprehensive enzyme kinetic studies in various stages, which require accurate measurements of reaction products.^5,8^ Also, real-time monitoring of carbohydrate concentrations in hydrolysates is crucial to achieving high yields and stable production cycles in the industry.^4^ However, the quantitative determination of these carbohydrates in hydrolysates is challenged by the insoluble nature of the biomass and the performed heterogeneous enzymatic reactions. Experimental approaches, such as quartz crystal microbalance, electrochemical biosensors, and isothermal titration calorimetry, have advanced recently.^4,9−12^ These analytical methods conquer the problem of insoluble polysaccharide substrates and are adequate to measure hydrolysis products under certain conditions. Among them, electrochemical biosensors have obtained the most attention for practical use, because they have high specificity and fast response, and are capable of continuous measurements in an aqueous suspension of any turbidity or coloration.

An amperometric cellobiose dehydrogenase (CDH)-based biosensor, which can replace colorimetric assays for the evaluation of cellulase activity, was first reported in 2001.^10^ More recently, glucose dehydrogenase and pyranose dehydrogenase were immobilized with redox mediators in carbon paste electrodes to study the transient kinetics of cellobiohydrolase or the hydrolyzation kinetics of crystalline cellulose by real-time measurement of cellobiose concentrations.^9,12,13^ Nevertheless, the biosensors in these studies required an anaerobic environment to conduct accurate measurements since the employed electron mediators (1,4-benzoquinone or 2,6-dichloroindophenol) donate electrons not only to dehydrogenases but also to oxygen. This limitation compromises their application in cellulolytic studies that include, for example, lignocellulolytic oxidoreductases (e.g., laccase or lytic polysaccharide monooxygenase), which utilize oxygen or oxygen-derived hydrogen peroxide as a co-substrate. However, these enzymes were found to act in concert with cellulases and are essential components in the overall biomass degradation process.^14−16^ Therefore, a cellobiose biosensor that is functioning accurately in the presence of oxygen is required.

On the other hand, the determination of glucose during the degradation of biomass is challenging due to the continual presence of the intermediate product cellobiose in the reaction suspension but essential to discriminate catalytic activities of cellulase blends.^2,17^ For example, the ratio of β-glucosidase in the cellulase cocktails needs to be adjusted according to the glucose production rate. In this work, we present two biosensors for the selective detection of cellobiose and glucose released from hydrolysis of phosphoric acid swollen cellulose (PASC) or milled corncob. A high-potential oxygen-insensitive redox mediator, the Os-complex-modified polymer poly(1-vinylimidazole-*co*-allylamine)-{[Os(2,2′-bipyridine)_2_Cl]Cl} (PVI-Os), is immobilized together with CDH to fabricate a cellobiose biosensor, which can measure the cellobiose concentration in the presence of oxygen.^18,19^ A glucose biosensor is built by combining a Pt catalyst for hydrogen peroxide detection with a surface layer of glucose oxidase (GOx).

## Experimental Section

### Reagents and Instruments

All chemicals were of high purity (>99%) and supplied by Sigma-Aldrich (St. Louis, MO, USA). Poly(ethylene glycol) diglycidyl ether (PEGDGE) having an average molecular weight distribution (*M*_n_) of 500 Da. PVI-Os was synthesized according to a published procedure.^18^ Electrochemical measurements were performed in a water-jacketed glass cell connected to a water bath using an Autolab PGSTAT204 potentiostat (Metrohm, The Netherlands) in 50 mM sodium acetate buffer of pH 5.0 (with or without a substrate) at 30.0 ± 0.2 °C. A magnetic stirrer operated at 600 rpm provided convective mass transport during amperometric measurements. A standard three-electrode configuration employed the CDH- or GOx-modified glassy carbon electrode (GCE, diameter 3.0 mm) as the working electrode, an Ag|AgCl (3 M KCl) reference electrode, and a platinum wire coil as an auxiliary electrode (BAS, USA). In the real-time measurement of hydrolysis reactions, the enzymes (cellulase, β-glucosidase, or CTec2) were delivered through a PEEK tube of 0.15 mm inner diameter connected to a syringe into the electrochemical cell. A DIONEX IC 5000 high-performance liquid chromatography (HPLC) system with a CarboPac PA100 column was used to determine cellobiose and glucose concentrations from hydrolysates during biosensor measurements.

### Enzymes

CDH (EC 1.1.99.18) from *Phanerochaete chrysosporium* was heterologously expressed in *Trichoderma reesei* and purified as described in a previous study.^20^ The RZ value (*A*_420_/*A*_280_) was 0.62 and indicated homogeneous enzyme preparation, exhibiting a specific activity of 17.5 U mg^–1^ determined with substrate cellobiose and 2,6-dichloroindophenol as the electron acceptor at pH 5.0. Cellulase from *T. reesei* (cellulase, 9012-54-8, 0.7 U mg^–1^), β-glucosidase from *Aspergillus niger* (9033-06-1, 0.75 U mg^–1^), Cellic CTec2 (cellulase, enzyme blend, ∼1.15 g/mL), and GOx (9001-37-0) from *A. niger* with a specific activity of 100–250 U mg^–1^ were all purchased from Sigma-Aldrich.

### Biosensors

Glassy carbon electrodes were polished on a polishing cloth with decreasing sizes of alumina suspensions (1, 0.3, and 0.05 μm), rinsed with Milli-Q water, and subsequently sonicated in water for 5 min before modification. For fabrication of the cellobiose biosensor, solutions of CDH (10 mg mL^–1^), PEGDGE (2 mg mL^–1^), and redox polymer PVI-Os (10 mg mL^–1^) were mixed in a volume ratio of 1:1:3 in 10 mM potassium phosphate buffer (pH 8.0). A 4.5 μL droplet of the mixture was dropped onto the GCE, followed by 12 h incubation at room temperature and high relative humidity (>80%). For the glucose biosensor, the polished GCE was first modified with a Pt catalyst by electrodeposition at −0.2 V versus Ag|AgCl for 150 s in 0.02 g L^–1^ chloroplatinic acid dissolved in 0.1 M H_2_SO_4_. A GOx/chitosan mixture was prepared by mixing GOx (10 mg mL^–1^) with a chitosan solution (2 mg mL^–1^) at a ratio of 1:1 and the final addition of glutaraldehyde (0.1%). Chitosan was dissolved in acetic acid, and the pH was adjusted to 4.5 by titration with sodium hydroxide solution. A 4.5 μL aliquot of the GOx/chitosan mixture was dropped onto the Pt catalyst-modified GCE and allowed to evaporate at room temperature overnight. For both biosensors, the modified electrodes were thoroughly rinsed with Milli-Q water and immersed in the agitated buffer solution to remove weakly adsorbed enzyme molecules for 5 min prior to use. For measurements during the degradation of corncob, both biosensors were covered with a dialysis membrane to restrain the enzymatic films from colliding with the substrate particles.^21^ A calibration was performed by consecutive titrations of 25 μL aliquots of cellobiose or glucose solutions into either the buffer solution or a 10 mg mL^–1^ corncob suspension.

### Enzymatic Hydrolysis of Cellulose

PASC was prepared from microcrystalline cellulose (an average particle size of 100 μm) using a reported method.^22^ Milled corncob was subjected to acid pretreatment before enzymatic hydrolysis. In short, 10 g of milled corncob (≤1.0 mm) was placed in a 250 mL glass bottle, suspended in 100 mL of 5.0% sulfuric acid, mixed for 1 h, and autoclaved at 121 °C for 30 min. The biomass was collected by centrifugation and washed with deionized water until the acid was fully removed. One part of the sample was dried in the oven at 85 °C overnight and then weighed to determine the mass loss. The other part was dispersed in deionized water and stored at −20 °C for hydrolysis experiments.

The measurements of cellobiose and glucose released from hydrolysis reactions were conducted in 15 mL suspensions of either PASC or milled corncob using the cellobiose and glucose biosensors at +0.25 and +0.45 V versus Ag|AgCl (3 M KCl), respectively. In a typical amperometric measurement, cellulase or CTec2 was injected to initiate hydrolysis when the background current reached a constant value over time at the applied potential. The scales of the enzyme dosage of cellulase, β-glucosidase, and CTec2 were all calibrated with the weight of the used biomass sample (PASC or corncob).

### Turbidimetric Measurements

Turbidimetric measurements can be used to determine semiquantitative concentrations of the cellulose suspension. Suspension samples of 1 mL were obtained from the electrochemical cell, where PASC hydrolysis was ongoing during chronoamperometry measurements with the biosensors. The optical density of PASC was determined at 620 nm using a single-beam UV–visible spectrophotometer (U-3000, Hitachi) with a built-in magnetic stirrer.^22^

### Sampling for HPLC Measurements

Each 0.5 mL sample was obtained from the hydrolysates of PASC or corncob at different time points. The active enzymes were instantly quenched by immersion in 95 °C for 10 min and removed from samples by centrifuging. The cellobiose and glucose concentrations in these samples were determined using high-performance anion-exchange chromatography with the pulsed amperometric detection (HPAE-PAD) method.

## Results and Discussion

### Characterization of Biosensors

CDH is composed of a larger catalytic dehydrogenase domain containing a flavin adenine dinucleotide (FAD) molecule as a cofactor and a smaller cytochrome domain containing heme *b*, which are connected through a flexible linker. The CDH immobilized on electrodes can catalyze the oxidation of cellobiose with electron mediators (the mediated electron transfer mode) or in a direct electron transfer mode.^19,23^

In previous studies, redox mediators such as 1,4-benzoquinone and 2,6-dichloroindophenol have been used to achieve a higher sensitivity.^12,13,18^ However, these rather low potential redox mediators donate electrons to molecular oxygen, which allows for accurate measurements only in an oxygen-free environment. Another disadvantage of the small molecular mediators is their leaking from the electrode, which can affect the followed reaction, especially in complex enzyme cocktails. The use of the redox polymer PVI-Os enables the electrical wiring of the FAD cofactor of CDH to the electrode (Figure S1).

**Figure 1:**
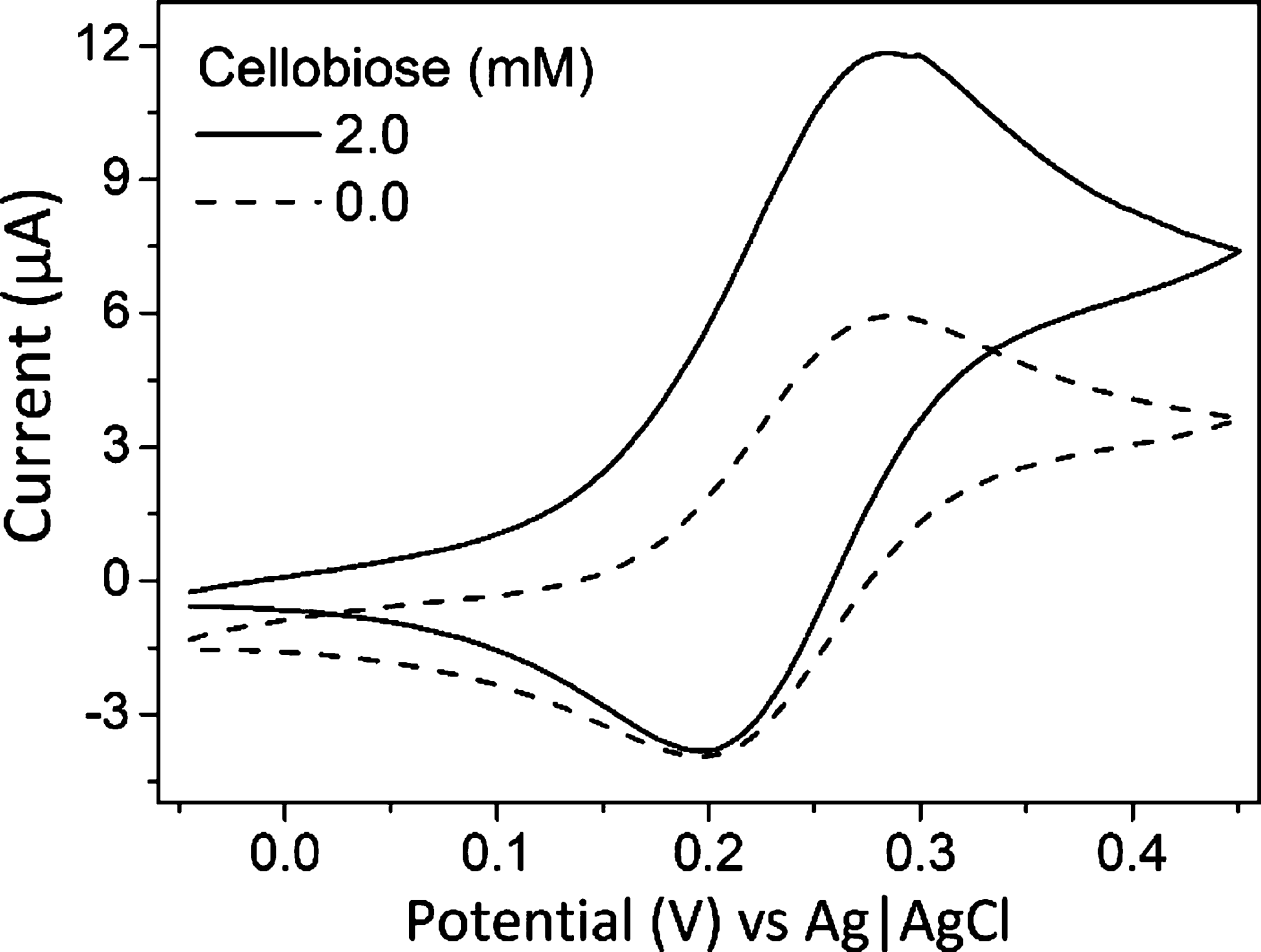
Characterization of the cellobiose biosensor. Cyclic voltammogram of the CDH biosensor measured with a scan rate of 50 mV s^–1^ in an acetate buffer without (dotted line) and with (solid line) 2 mM cellobiose.

It also alleviates mediator leaking because of a strong coordination of the Os complex centers to the polymer chain.^24^ Cyclic voltammetry was performed to characterize the electrocatalytic activity and determine the optimal potential for the cellobiose biosensor. The cyclic voltammogram in the absence of cellobiose(Figure 1, dotted line) showed a pair of chemically reversible current peaks with a midpoint potential of +0.22 V versus Ag|AgCl (3 M KCl) at pH 5.0, which was attributed to the Os(II)/Os(III) interconversion.^25^ Addition of 2 mM cellobiose generated a rise of the anodic current around the midpoint potential of the polymer-bound Os complex centers. Therefore, cellobiose biosensors have been biased at +0.25 V to perform all the chronoamperometry measurements. This potential is slightly above the midpoint potential of the Os complex and thus ensures a high thermodynamic driving force for the electron transfer but ensures at the same time that no undesired side reaction at high potential occurs.

**Figure 2:**
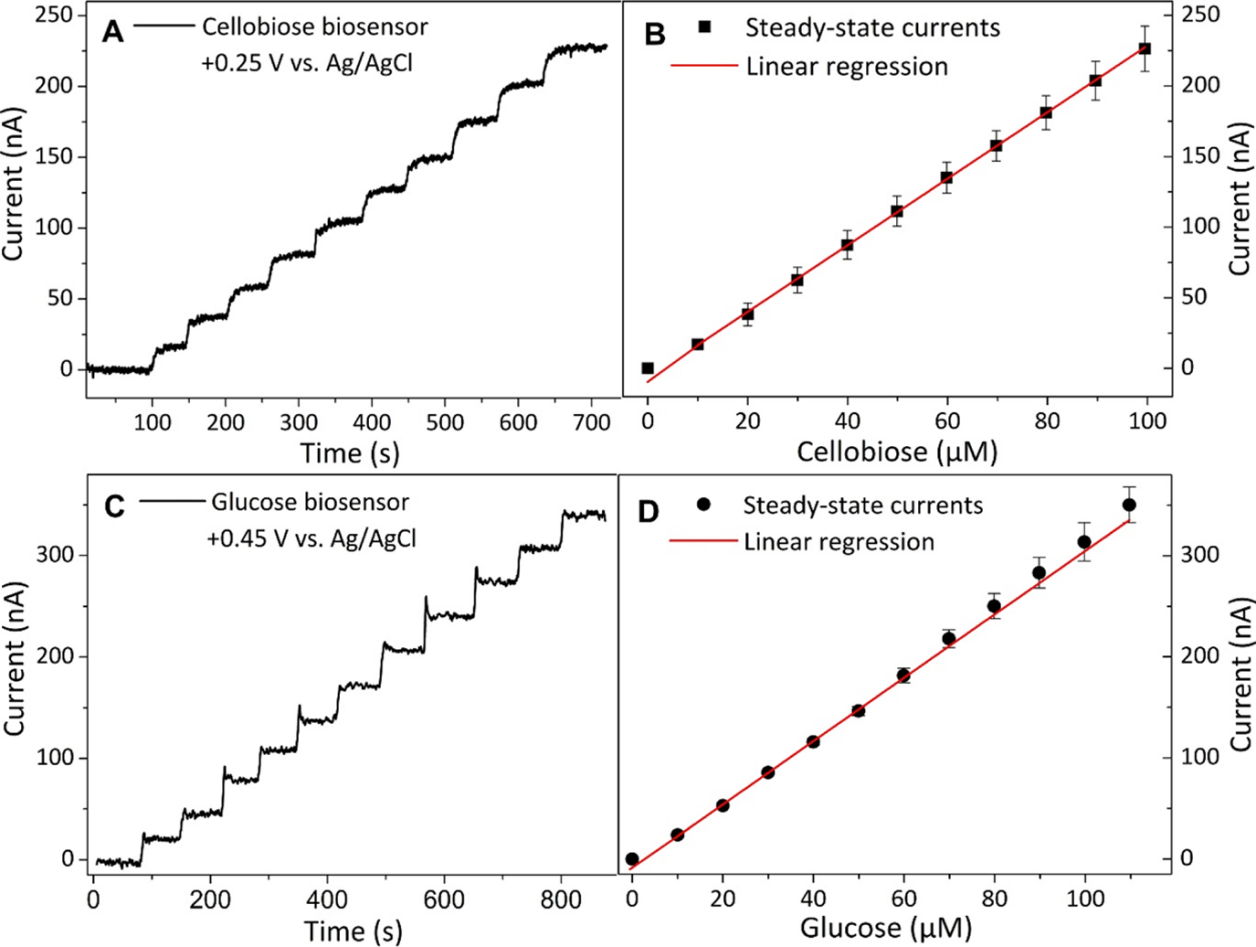
Calibration of cellobiose and glucose biosensors. The left panels show the amperometric response of the cellobiose (A) and the glucose biosensor (C) at increasing concentrations of cellobiose or glucose, respectively, at 30 °C in agitated 50 mM sodium acetate buffer, pH 5.0. The right panels show the corresponding calibration plots of the cellobiose (B) and glucose biosensors (D). The error bars indicate the standard deviation of current values measured from three different biosensors. The steady-state current of each plot is corrected for the background current.

Figure 2A shows that the Faradaic currents reached stable plateaus within 5 s for each addition of 10 μM cellobiose. The steady-state current linearly increased with the cellobiose concentration up to 100 μM with a sensitivity of 2.39 nA μM^–1^ and a detection limit (S/N = 3) of 2.55 μM (Table S1). A relatively low potential of +0.45 V was used for our glucose biosensor owing to the platinum black catalyst.^26^ The response of chronoamperometry and the corresponding calibration curve are shown in Figure 2C,D. Up to 110 μM, the increase followed a linear trend with a sensitivity of 3.23 nA μM^–1^ and a detection limit (S/N = 3) of 0.65 μM (Table S2).

The conversion of cellulose into fermentable sugar generally requires two sequential steps. First, cellulose is hydrolyzed to cellobiose by cellulase. Subsequently, cellobiose is converted by β-glucosidases into glucose.^2,25^ In the whole hydrolytic process, cellobiose and glucose coexist in the reaction system. Thus, the selectivity of the biosensors greatly affects the accuracy of the measured analyte concentrations in this complex matrix.

**Figure 3:**
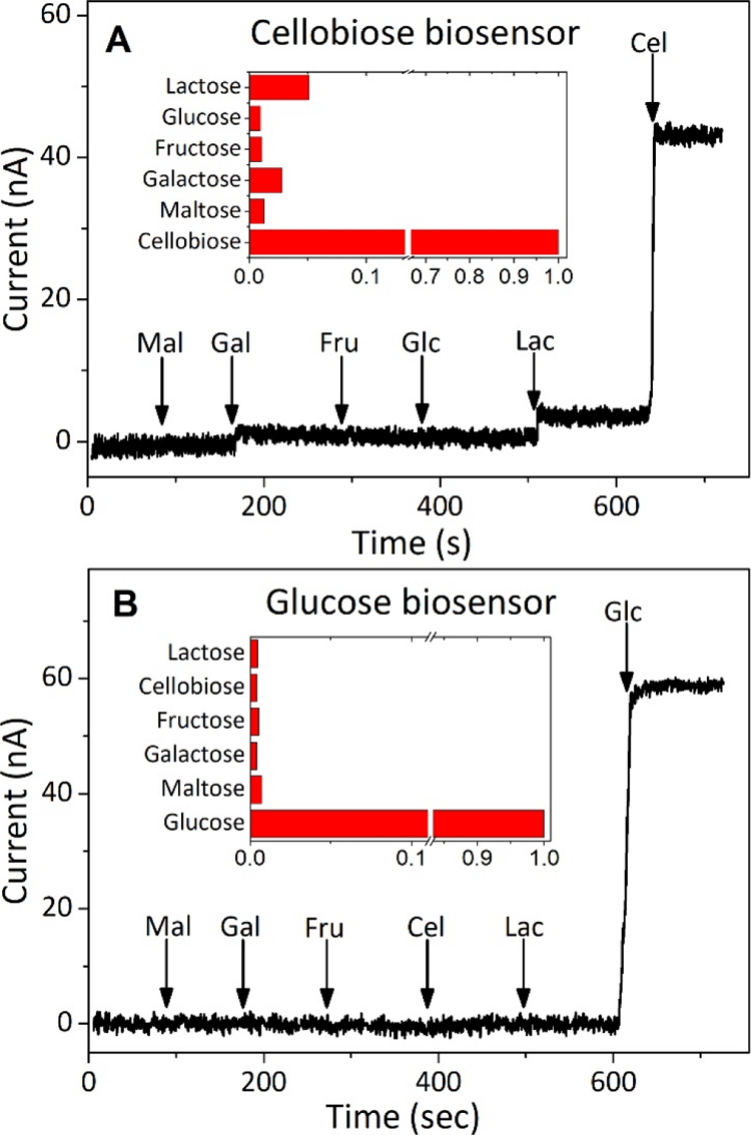
Selectivity test of the cellobiose and glucose biosensors. Amperometric response of cellobiose (A) and glucose (B) biosensors to respective analytes in the presence of potential interfering substrates. The concentrations of interfering sugars (maltose, Mal; galactose, Gal; fructose, Fru; glucose, Glu; lactose, Lac; and cellobiose, Cel) are all 50 μM, while the concentration of the target analyte (cellobiose in A and glucose in B) is 20 μM. Arrows indicate the additions of the analyte or interfering substrates.

For the cellobiose biosensor, the interference currents from glucose (Glc), fructose (Fru), and maltose (Mal) conversion were less than 2.0% of the signal, while lactose (Lac) and galactose (Gal) generated 5.3 and 2.8% of the additional current, respectively (Figure 3A). Except for that from lactose, a disaccharide very similar to cellobiose but not present in lignocellulose samples, the interference from other reducing sugars was negligible (<3.0%). For the glucose biosensor, the currents induced by all the interfering substrates were smaller than 1.2% (Figure 3B). This mediator-less glucose biosensor design had a much higher selectivity for glucose than a preliminary-tested PVI-Os-wired GOx electrode, which showed a very high signal for interfering cellobiose. Therefore, highly specific detection of cellobiose and glucose in hydrolysates of plant biomass can be accomplished by a combination of the two biosensors.

**Figure 4:**
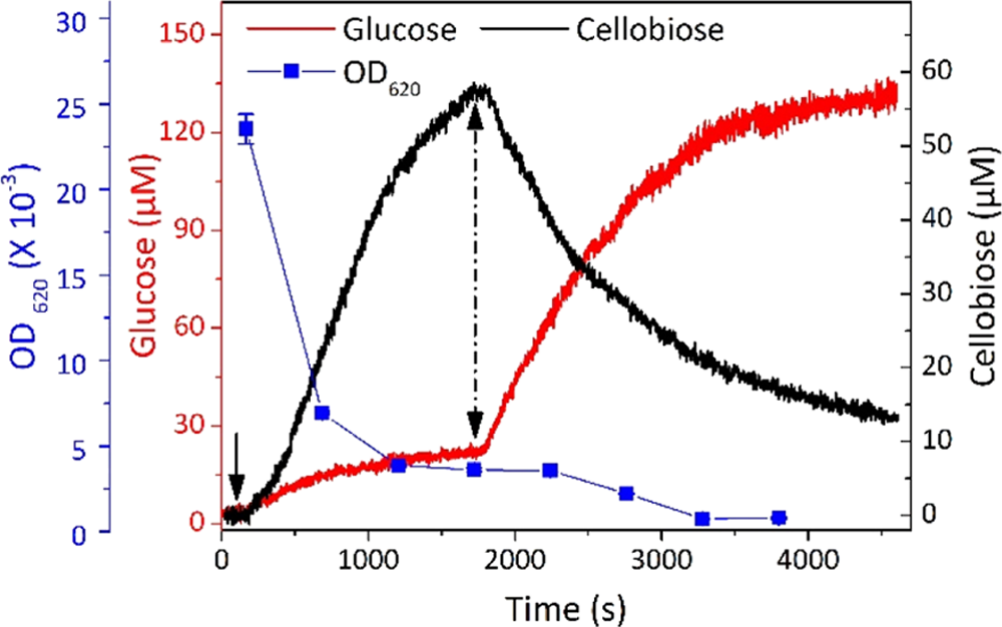
Continuous monitoring of PASC hydrolysis using both biosensors and a photometer. The arrows indicate the addition of 1.0% cellulase at 100 s and 2.0% β-glucosidase at 1700 s. The substrate was constantly stirred by a magnetic bar in 50 mM sodium acetate buffer, pH 5.0, at 30 °C.

In the first hydrolysis experiment, we employed PASC because of its low crystallinity, which provided better access for cellulase enzymes and also lacked potentially interfering compounds such as hemicelluloses and lignin-derived phenols in corncob. In the initial stage, 1.0% cellulase was administrated to 0.1 mg mL^–1^ agitated PASC suspension after a stable background current was obtained. After about 60 s of incubation, the currents recorded by both cellobiose and glucose biosensors were increased, indicating that products from PASC hydrolysis were detected. Cellobiose as the main product was rapidly produced, reaching a concentration of 58.0 ± 1.8 μM after 1600 s of hydrolysis (Figure 4). The cellobiose release proceeded at a fast rate between 160 and ∼1200 s, corresponding to the distinctive initial burst of endoglucanase and exoglucanase within cellulase.^27^

Within 1600 s, 86.8% of PASC was converted according to the reduction of OD_620_ obtained from turbidimetric measurements, indicating that the insoluble cellulose fibers were rapidly depolymerized to soluble cellobiose (blue squares in Figure 4). The glucose concentration calculated from the measured current increased to 21.5 ± 2.2 μM in this period. This slow increase of the glucose concentration is due to the fact that a very small portion of β-glucosidases is also present in cellulase from *T. reesei*.^28^

The second stage started with the injection of 2% β-glucosidase. About 10 s after the injection, the cellobiose concentration dropped quickly (2.2 μM min^–1^), while the glucose concentration increased proportionally (5.3 μM min^–1^) in the period of 1700–2300 s. Then, the conversion of cellobiose slowed down gradually to final concentrations of 14 ± 1.6 μM cellobiose and 128.7 ± 4.3 μM glucose at 4600 s. In this stage, OD_620_ was further reduced by 9.2% to the final value of 0.003 at 3200 s, indicating nearly full hydrolysis of PASC. The stoichiometry of the conversion from cellobiose to glucose is 1:2, which was reflected by the measured cellobiose (58.0 μM) and glucose (107.2 μM) concentrations. Subsequent experiments showed that the conversion rate of cellobiose increased with the increasing dosage of β-glucosidase administered (Figure S2).

The above results demonstrate the ability of the two biosensors to measure cellobiose and glucose concentrations during the hydrolysis of PASC. To investigate a more complex biomass sample, milled corncob, a common raw material in the biofuel industry, was selected. Because of the frequent collision of corncob particles (<1.0 mm) in agitated buffer solution, both biosensors were covered with a dialysis membrane with a pore size of 15 nm to retain the coating of enzymes and redox polymers during measurements.

**Figure 5:**
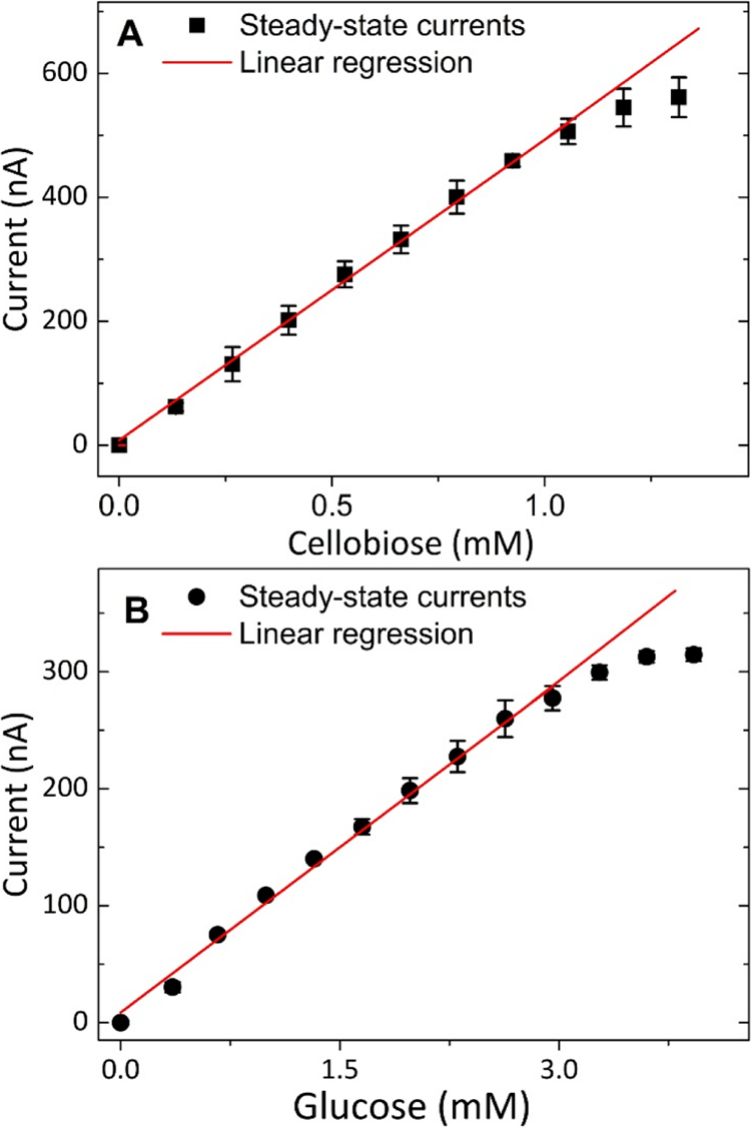
Calibration of membrane-covered cellobiose and glucose biosensors. The plots of the steady-state currents vs cellobiose (A) and glucose (B) concentrations were obtained in 50 mM acetate buffer, pH 5.0, containing 10 mg mL^–1^ milled corncob at 30 °C. Error bars show the standard deviations of triplicate measurements. Each measured current was corrected for the background current.

The membrane coverage changed the biosensor properties regarding the response time, sensitivity, and linear detection range. A new calibration for the membrane-covered biosensors was obtained by titrating the substrates in a buffered solution containing the milled corncob.^29^ The sensitivity of the membrane-covered cellobiose biosensor decreased to 0.48 nA μM^–1^, but its linear detection range extended up to 1 mM (Figure 5A). For the membrane-covered glucose biosensor, the sensitivity was reduced to 0.094 nA μM^–1^, and the linear detection range increased to ∼3 mM (Figure 5B). These changes can be explained by the mass transfer restriction exerted on the substrates by the membrane. The response time of both biosensors increased significantly (from 5 to 22 s for the cellobiose biosensor and from 3 to 11 s for the glucose biosensor) when covered with the membrane. This long response time has to be considered if the pre-steady-state kinetics are assessed but does not affect our measurements in steady-state reactions.

To mimic an industrial hydrolysis process, the CTec2 cellulase blend was used to hydrolyze the corncob slurry. CTec2 can directly decompose a wide variety of lignocellulosic biomass feedstocks into fermentable sugars. Similar to the first experiment, 2% CTec2 was injected into the stirred corncob slurry (Figure 5A). It is notable that at the applied potential of +0.45 V versus Ag|AgCl (3 M KCl), a constant oxidative Faradaic current from components of the cellulase blend was detected in the absence of corncob for over 1 h (Figure S3). However, this interference was not observed at the polarization of +0.25 V versus Ag|AgCl (3 M KCl) for the cellobiose biosensor. To eliminate this interference, the recorded currents of the glucose biosensors were corrected for the current induced by CTec2.

**Figure 6:**
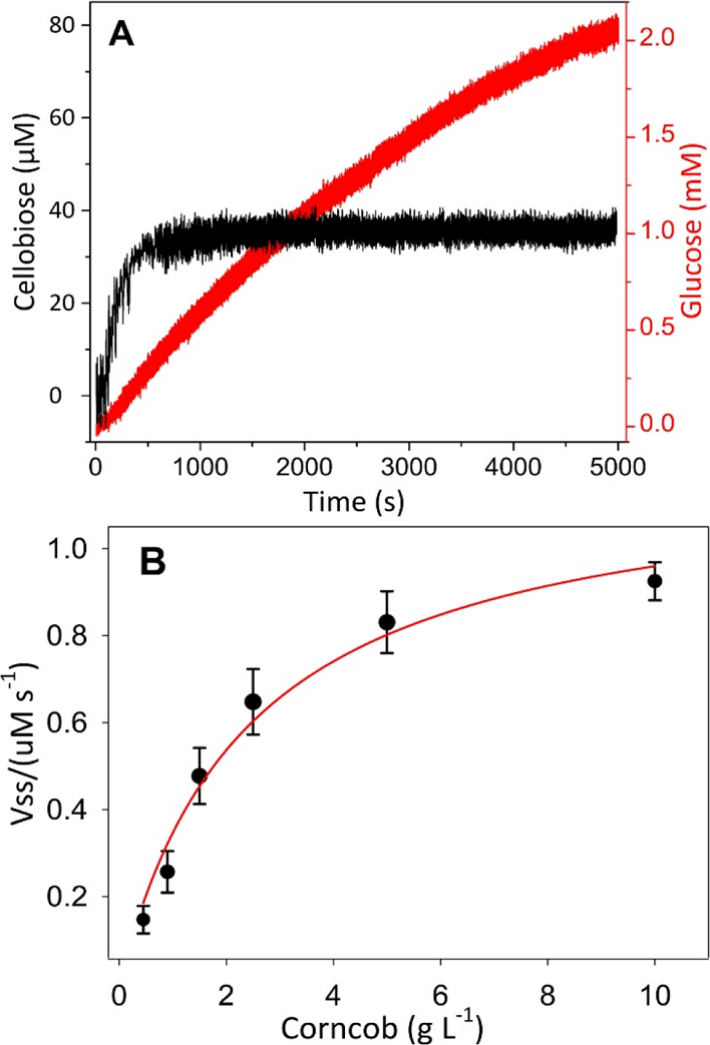
Study of corncob degradation using the biosensors. (A) Real-time measurements of glucose and cellobiose formation in the 5 g L^–1^ corncob suspension which was degraded by 10.0% CTec2. (B) The plot of the reaction rate vs substrate concentration approximates a hyperbolic function. The substrate was constantly stirred by a magnetic bar in 50 mM sodium acetate buffer, pH 5.0, at 30 °C.

During corncob hydrolysis, the glucose concentration increased continuously and reached ∼2.15 mM after 5000 s with an average production rate of 25.8 μM min^–1^ under the given conditions. Meanwhile, the cellobiose concentration reached ∼34.3 μM after 530 s and remained constant until the end (Figure 6A). This could indicate a steady-state equilibrium of cellobiose formation and its further conversion into glucose by β-glucosidase. The glucose biosensor was also used to conduct measurements in the hydrolysis processes with varied loadings of milled corncob. The formation rate of glucose dependent on the increasing dosage of corncob with a constant enzyme load was calculated from each experiment (Figure S4). The steady-state hydrolysis rate (calculated between 1900 and 2000 s in Figure S4) was plotted as a function of the substrate dosage (Figure 6B). Minimum least squares regression showed a relatively good fit to a hyperbolic function (*R*^2^ = 0.98), which predicted the cellulolytic activity of the employed enzymes based on the added biomass concentration and the initially available access sites for exoglucanases and endoglucanases present in CTec2.^5^ Extrapolation to an infinite substrate concentration showed a maximum rate of 1.2 μmol s^–1^, and 50% of the maximum hydrolysis rate could be achieved at 2.45 g L^–1^ milled corncob.

**Figure 7:**
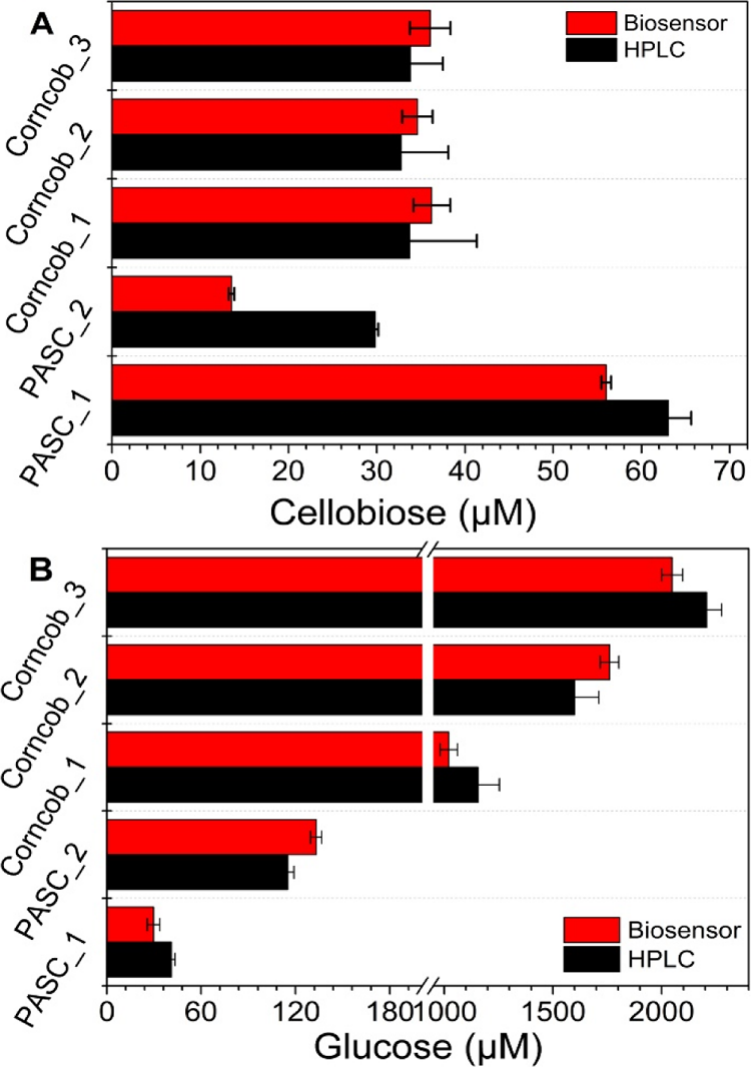
HPLC analysis. Comparison of cellobiose (A) and glucose (B) concentrations obtained from biosensors (red bars) and HPAE-PAD (black bars).

The results of biosensors measurements were compared to data from HPAE-PAD. The samples Corncob_1, Corncob_2, and Corncob_3 were obtained at 1800, 3600, and 5000 s, respectively, from the hydrolysis of corncob (Figure 7A), and the samples PASC_1 and PASC_2 were obtained from the hydrolysis of PASC at 1700 s (before the addition of β-glucosidase) and 4500 s, respectively (Figure 4). The cellobiose (Figure 7A) and glucose (Figure 7B) concentrations of corncob samples showed a ≤6.0% difference, which is within the current noise of chronoamperometry (error of the method). However, the observed biosensor data for the formed glucose in PASC_1 and the formed cellobiose in PASC_2 differed from the HPLC analysis by 31.8 and 43.4%, respectively. The reason is probably that CDH and GOx are inactive against the α-anomeric forms of their substrates and therefore specifically detect the β-anomer of cellobiose or glucose. In sample PASC_1, glucose was produced at the beginning, and accumulated glucose already underwent some mutarotation, which generated α-glucose, whereas in sample PASC_2, cellobiose was accumulated to a relatively high level, which can be confirmed by the slow increase in the current after ∼30 min (Figure S1). Once β-glucosidase was introduced, the equilibrium was interrupted as the rate of β-anomer consumption is much faster than that of its isomer conversion.^30^ As a result, the excess α-anomer could be detected by HPAE-PAD but not by the cellobiose biosensor. In all, the effect of exogenous substrate (cellobiose or glucose) accumulation, which can disturb the dynamics of mutarotation, should be considered in real-time measurements. Importantly, the comparison shows that the lignin and other potentially electroactive compounds in the milled corncob did not influence the biosensor and allows an accurate and time-resolved measurement of biomass hydrolysis using multienzyme preparations.

## Conclusions

A CDH-based biosensor and a GOx-based biosensor were fabricated to specifically detect cellobiose and glucose, which are released during the hydrolysis of cellulose and milled corncob, respectively. Their high sensitivity and selectivity allow the *in situ* study of biomass hydrolysis and parallel detection of the reaction intermediate cellobiose and the final product glucose in the presence of each other. A dialysis membrane protects the biosensors from damage by corncob particles and increases the linear range of the biosensors. A commercial cellulase blend (CTec2) was used to hydrolyze the corncob slurry, and the biosensors recorded measurements up to 5000 s. Based on the obtained data, the steady-state concentration of cellobiose and the production rate of glucose could be determined. The obtained results from the biosensors were supported by HPLC analysis, which also showed that the biosensors were not affected by electroactive substances in the biomass such as lignin-derived phenols. This study demonstrates that biosensors can be a promising tool for the investigation of fungal enzymes bound to biomass.

## Supporting Information Available

Schematic illustration of the catalytic mechanism of the two biosensors; analytical parameters of the cellobiose and glucose biosensors used for the investigation of hydrolysis of cellulose samples; study of ASC hydrolysis with varying dosages of β-glucosidase using both biosensors; chronoamperometry response of the glucose biosensor during the hydrolysis of corncob with commercial CTec2; and study of the hydrolysis of varying corncob loadings with glucose biosensors (PDF)
