# Supplementary material for: Real-Time Measurement of Cellobiose and Glucose Formation during Enzymatic Biomass Hydrolysis
Source: Anal Chem. 2021 May 20;93(21):7732–8. doi: 10.1021/acs.analchem.1c01182 (PMC8173519; doi:10.1021/acs.analchem.1c01182)
Supplement: Supplementary file 1 — ac1c01182_si_001.pdf [file ac1c01182_si_001.pdf]

Supplementary Materials

Real-time measurement of cellobiose and glucose formation during enzymatic biomass hydrolysis

Hucheng Chang[1], Lena Wohlschlager[1], Florian Csarman[1], Adrian Ruff[2,3], Wolfgang Schuhmann[2], Stefan Scheiblbrandner[1], Roland Ludwig[1]*

[1]Biocatalysis and Biosensor Laboratory, Department of Food Science and Technology, BOKU–University of Natural Resources and Life Sciences, Muthgasse 18, 1190 Vienna, Austria

[2]Analytical Chemistry–Center for Electrochemical Sciences (CES), Faculty of Chemistry and Biochemistry, Ruhr University Bochum, Universitätsstraße 150, 44780 Bochum, Germany

[3] Present Address: PPG Packaging Analytical Labs, PPG Deutschland Business Support GmbH, Erlenbrunnenstr. 20, 72411 Bodelshausen, Germany.

* Corresponding Author: roland.ludwig@boku.ac.at

Table of Contents

S1. Schematic illustration of catalytic mechanism of the two biosensors 2

S2. Analytical parameters of cellobiose biosensors..3

S3. Analytical parameters of glucose biosensors..3

S4. Analytical parameters of membrane-covered cellobiose biosensors..4

S5. Analytical parameters of membrane-covered glucose biosensors..4

S6. Study of PASC hydrolysis with varying dosage of β-glucosidase using both biosensors..........5

S7. Chronoamperometry response of glucose biosensor during hydrolysis of corncob with commercial CTec2..5

S8. Study of the hydrolysis of varying corncob loading with glucose biosensors............................6

S1. Schematic illustration of catalytic mechanism of the two biosensors

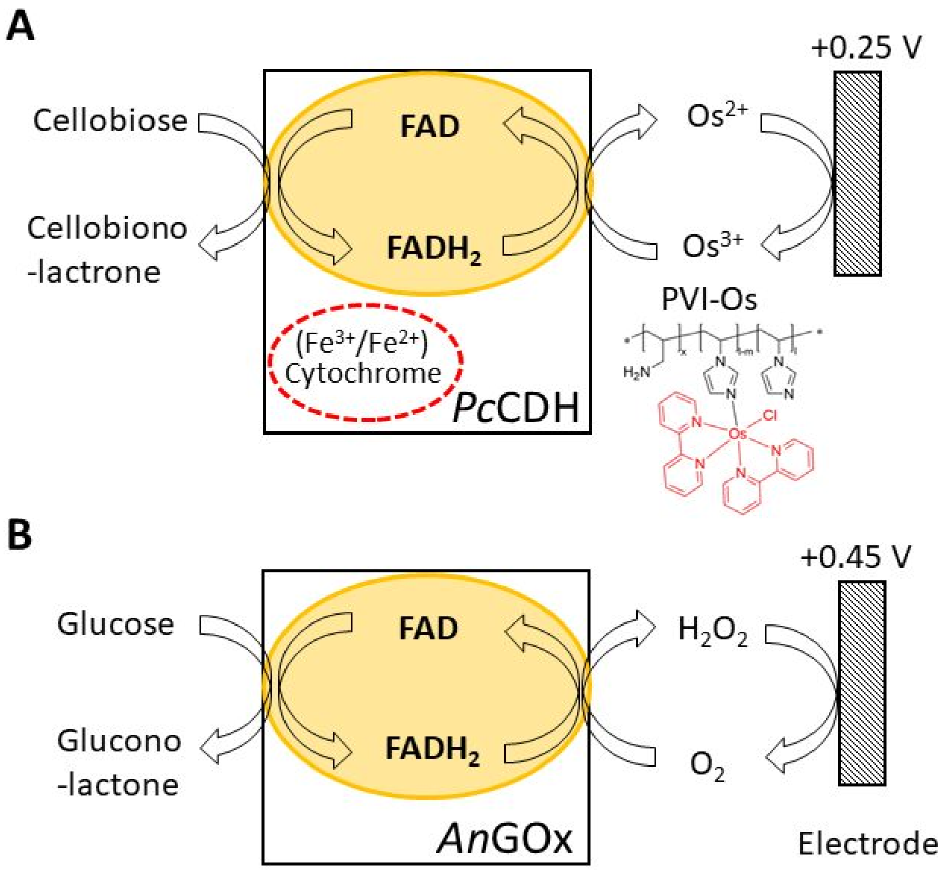

Figure S1 Schematic illustration of the catalytic mechanism of cellobiose (A) and glucose (B) biosensors.

S2. Analytical parameters of cellobiose biosensors

Table S1 Analytical parameters of three independent cellobiose biosensors. The amperometric measurements were performed in agitated 50 mM acetate buffer, pH 5.0, at 30 °C with titration of cellobiose.

Cellobiose biosensor				
Parameter	Sensor 1	Sensor 2	Sensor 3	Average
Sensitivity (nA μM^{-1})	2.67 (± 0.04)	2.24 (± 0.02)	2.25 (± 0.03)	**2.39 (± 0.03)**
R^2	>0.999	>0.996	>0.999	**>0.998**
Noise, 60 sec (nA)	2.77 (± 0.05)	1.57 (± 0.02)	1.82 (± 0.04)	**2.05 (± 0.04)**
Noise/ Sensitivity (µM)	1.04	0.70	0.81	**0.85**
Detection limit (3σ) (µM)	3.12	2.10	2.43	**2.55**
Linear detection range (µM)	100	95	100	**98.33**
Response time, $t_{95\%}$ (s)	5.2	6.0	5.0	**5.10**

S3. Analytical parameters of glucose biosensors

Table S2 Analytical parameters of three independent glucose biosensors. The amperometric measurements were performed in agitated 50 mM acetate buffer, pH 5.0, at 30 °C with titration of glucose.

Glucose biosensor				
Parameter	Sensor 1	Sensor 2	Sensor 3	Average
Sensitivity (nA μM^{-1})	3.65 (± 0.04)	3.01 (± 0.03)	3.04 (± 0.03)	**3.23 (± 0.03)**
R^2	>0.998	>0.996	>0.999	**>0.999**
Noise, 60 sec (nA)	1.07 (± 0.07)	0.56 (± 0.03)	0.53 (± 0.06)	**1.65 (± 0.07)**
Noise/ Sensitivity (µM)	0.29	0.19	0.17	**0.22**
Detection limit (3σ) (µM)	0.87	0.57	0.51	**0.65**
Linear detection range (µM)	110	110	110	**110**
Response time, $t_{95\%}$ (s)	3.2	2.8	3.3	**3.1**

S4. Analytical parameters of membrane-covered cellobiose biosensors

Table S3. Analytical parameters of three independent membrane-covered cellobiose biosensors. The amperometric measurements were performed in agitated 50 mM acetate buffer, pH 5.0, containing 10 mg mL^{-1} milled corncob at 30 °C with titration of cellobiose.

Membrane-covered cellobiose biosensor				
Parameter	Sensor 1	Sensor 2	Sensor 3	Average
Sensitivity (nA mM^{-1})	483.72 (± 12.7)	481.24 (± 9.1)	488.75 (± 8.4)	**484.57 (± 10.7)**
R^2	>0.999	>0.998	>0.999	**>0.998**
Noise, 60 sec (nA)	2.56 (± 0.1)	3.87 (± 0.06)	2.32 (± 0.13)	**2.92 (± 0.1)**
Noise/ Sensitivity (µM)	5.30	8.04	4.75	**6.03**
Detection limit (3σ) (µM)	15.90	24.12	14.25	**18.08**
Upper limit (linear range) (mM)	1.13	1.15	1.19	**1.17**
Response time, $t_{95\%}$ (s)	25.3	24.5	23.0	**24.2**

S5. Analytical parameters of membrane-covered glucose biosensors

Table S4. Analytical parameters of three membrane-covered glucose biosensors. The amperometric measurements were performed in 50 mM acetate buffer, pH 5.0, containing 10 mg mL^{-1} milled corncob at 30 °C with titration of glucose.

Membrane-covered glucose biosensor				
Parameter	Sensor 1	Sensor 2	Sensor 3	Average
Sensitivity (nA mM^{-1})	95.54 (± 2.51)	93.27 (± 1.93)	93.12 (± 4.34)	**93.96 (± 2.93)**
R^2	>0.999	>0.997	>0.996	**>0.997**
Noise, 60 sec (nA)	4.37 (± 0.30)	4.57 (± 0.16)	3.82 (± 0.22)	**4.26 (± 0.23)**
Noise/ Sensitivity (µM)	45.74	49.00	41.02	**45.25**
Detection limit (3σ) (µM)	137.22	147.00	123.06	**135.76**
Linear detection range (mM)	2.96	3.03	2.98	**2.99**
Response time, $t_{95\%}$ (s)	12.0	11.5	12.6	**12.1**

S6. Study of PASC hydrolysis with varying dosage of β-glucosidase using both biosensors

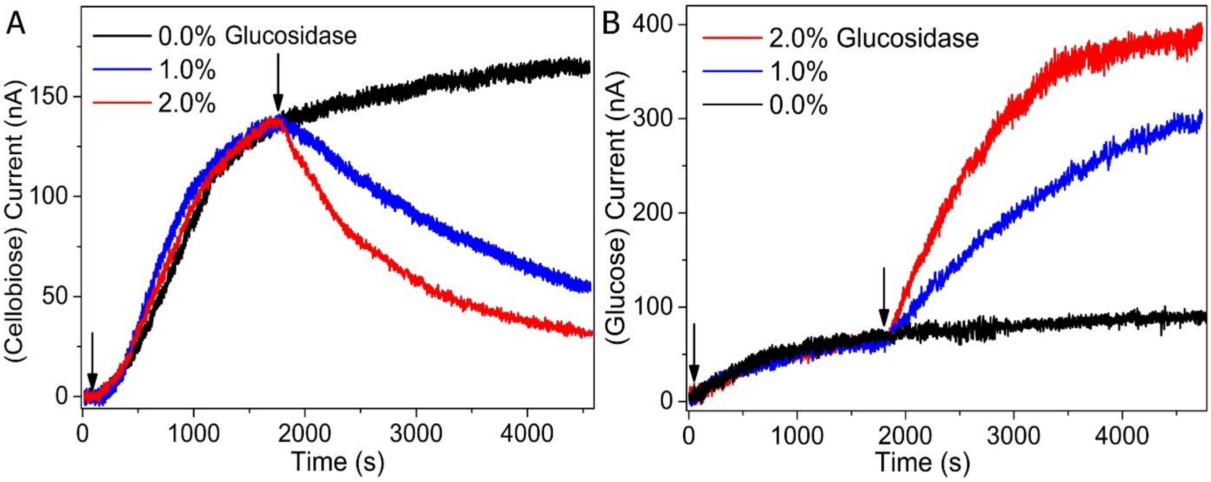

Figure S2 Chronoamperometry measurements of cellobiose (A) and glucose (B) formation during the hydrolysis of PASC by 1.0% Cellulase and varying dosage of β-glucosidase in the agitated 0.1 M sodium acetate buffer, pH 5.0, at 30 °C. The arrows indicate the time points upon addition of cellulase or β- glucosidase.

S7. Chronoamperometry response of glucose biosensor during hydrolysis of corncob with commercial CTec2

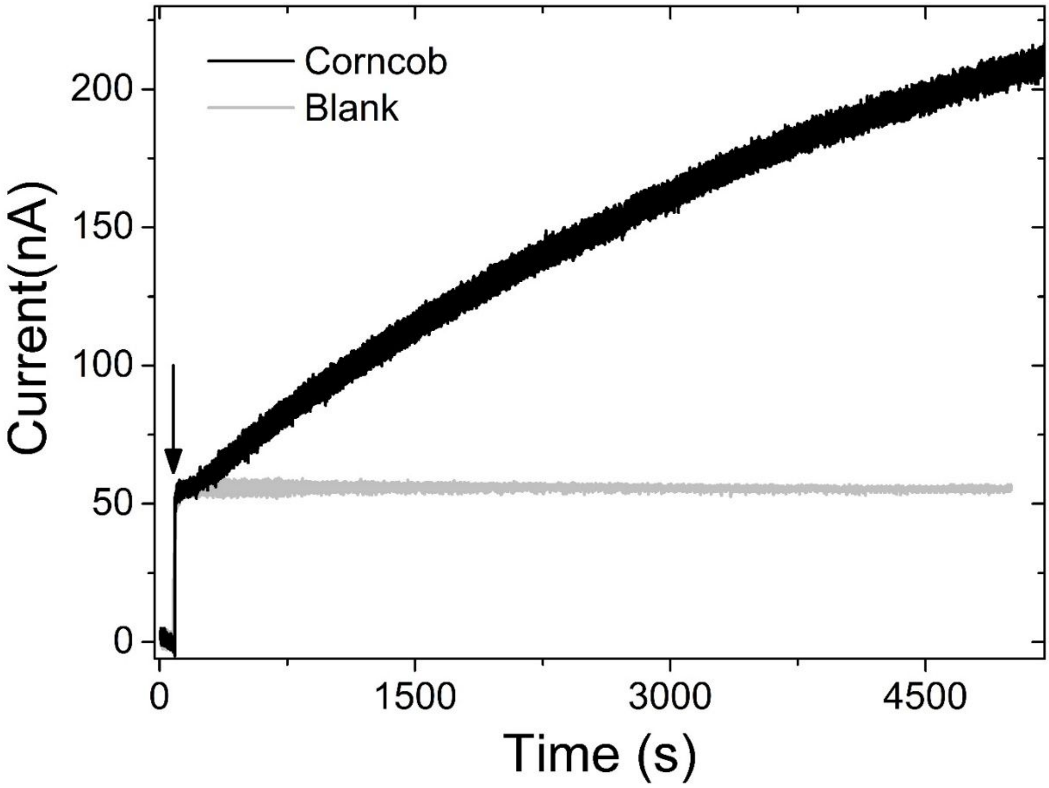

Figure S3 Chronoamperometry response (at +0.45 V) of the glucose biosensor to 3 μL-cellulase blend (CTec2) without and with the presence of 2 g L^{-1} milled corncob in 0.1 M agitated sodium acetate buffer, pH 5.0, at 30 °C. The arrow indicates the addition of 3 μL of CTec2.

S8. Study of the hydrolysis of varying corncob loading with glucose biosensors

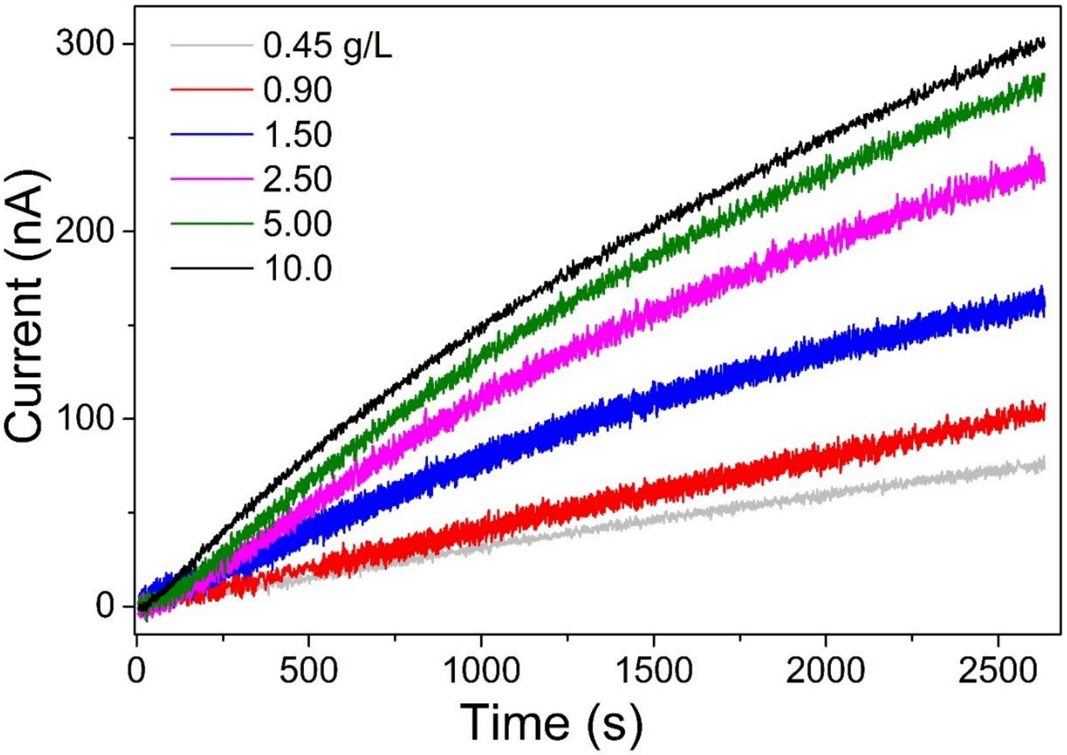

Figure S4 Chronoamperometry measurements of glucose formation during the hydrolysis of varying dosage of milled corncob with the fixed dosage (13 µL) of cellulase blend CTec2 in the agitated 0.1 M sodium acetate buffer, pH 5.0, at 30 °C. The interference currents of cellulase blend for each plot have been subtracted and not shown. Therefore, the hydrolysis in all the measurements started from the origin in the graph.
